# Plasma proteomics reveals molecular overlap between physical activity and dementia risk

**DOI:** 10.1093/braincomms/fcag287

**Published:** 2026-07-21

**Authors:** Rowan Saloner, Emily W Paolillo, Anna M VandeBunte, Claire J Cadwallader, Coty Chen, Brian T Steffen, David A Bennett, Bradley F Boeve, Howard J Rosen, Adam L Boxer, Joel H Kramer, Kaitlin B Casaletto

**Affiliations:** Memory and Aging Center, Department of Neurology, Weill Institute for Neurosciences, University of California San Francisco, San Francisco, CA 94158, USA; Memory and Aging Center, Department of Neurology, Weill Institute for Neurosciences, University of California San Francisco, San Francisco, CA 94158, USA; Memory and Aging Center, Department of Neurology, Weill Institute for Neurosciences, University of California San Francisco, San Francisco, CA 94158, USA; Memory and Aging Center, Department of Neurology, Weill Institute for Neurosciences, University of California San Francisco, San Francisco, CA 94158, USA; Memory and Aging Center, Department of Neurology, Weill Institute for Neurosciences, University of California San Francisco, San Francisco, CA 94158, USA; Division of Computational Health Sciences, Department of Surgery, University of Minnesota, Minneapolis, MN 55455, USA; Rush Alzheimer’s Disease Center, Rush University Medical Center, Chicago, IL 60612, USA; Department of Neurology, Mayo Clinic, Rochester, MN 55905, USA; Memory and Aging Center, Department of Neurology, Weill Institute for Neurosciences, University of California San Francisco, San Francisco, CA 94158, USA; Memory and Aging Center, Department of Neurology, Weill Institute for Neurosciences, University of California San Francisco, San Francisco, CA 94158, USA; Memory and Aging Center, Department of Neurology, Weill Institute for Neurosciences, University of California San Francisco, San Francisco, CA 94158, USA; Memory and Aging Center, Department of Neurology, Weill Institute for Neurosciences, University of California San Francisco, San Francisco, CA 94158, USA

**Keywords:** exercise, angiogenesis, dementia, proteomics, biomarkers

## Abstract

Physical activity (PA) is a modifiable lifestyle behaviour associated with lower dementia risk; however, molecular pathways bridging PA-related dementia prevention are poorly understood. We leveraged large-scale plasma proteomics to identify biological signatures of objectively monitored PA and cognitive ageing in functionally intact older adults, cross-validated these signatures in independent exercise cohorts and tested associations with both symptomatic and presymptomatic stages of neurodegeneration across multiple Alzheimer’s disease and related dementias (ADRD) cohorts. We analysed large-scale plasma proteomics data (SomaScan 7k) across three cohorts including naturalistic, objective PA monitoring (University of California, San Francisco Brain Aging Network for Cognitive Health cohort, *n* = 65), self-reported PA (Atherosclerosis Risk In Communities study, *n* = 10 644) and PA intervention (Health Risk Factors, Exercise Training and Genetics study, *n* = 654). Differential regression models examined individual protein correlates of PA, adjusting for age and sex. Weighted gene co-expression network analysis assembled proteins into unbiased modules of protein co-expression, which were annotated for gene ontology and cell-type enrichment. To test clinical relevance to ADRD, we examined PA-related protein levels across-cohorts of symptomatic Alzheimer’s disease and Parkinson’s disease (Stanford Alzheimer’s Disease Research Center), as well as frontotemporal dementia-spectrum disorders (ARTFL/LEFFTDS Longitudinal Frontotemporal Lobar Degeneration consortium). PA-related plasma proteins were also tested as predictors of antemortem cognitive change and post-mortem brain tissue mass spectrometry proteomic signatures in brain donors from the Religious Orders Study and Rush Memory and Aging Project (ROSMAP) cohort. Differential regression and network analyses identified PA plasma proteomic signatures linked to cell adhesion/extracellular matrix (ECM), immune response and lipid metabolism. Protein co-expression module M12 ECM/neurodevelopment harboured growth factor, cell adhesion and vascular remodelling proteins that (i) were positively associated with PA across exercise cohorts, (ii) positively associated with cognitive function and (iii) negatively associated with Alzheimer’s disease, Parkinson’s disease and frontotemporal dementia. Furthermore, M12 was enriched for proteins from Alzheimer’s disease risk genes and antemortem plasma abundance of anthrax toxin receptor cell adhesion molecule 2 (ANTXR2), an M12 ‘hub’ protein and top PA hit across-cohorts, forecasted longitudinal global cognitive decline and post-mortem brain tissue signatures of synaptic function and proteolysis in ROSMAP. Collectively, our integrated systems biology analysis of six independent plasma proteomic datasets facilitated discovery and validation of blood-detectable molecular signatures of PA and neurodegenerative disease, including PA-related proteins with clinical and biological relevance to early stages of disease. Circulating levels of PA-related proteins reflecting ECM biology (e.g. ANTXR2) may represent key molecular targets for dementia prevention.

## Teaser

Saloner *et al*. identify immunovascular and neuroplasticity plasma proteomic signatures of physical activity (PA) linked to neurodegeneration. Higher plasma anthrax toxin receptor 2 is related to greater PA, cognitive resilience and greater post-mortem synaptic integrity. Plasma proteomics may capture angiogenic pathways and molecules through which PA supports brain health.

## Graphical Abstract

**Graphical Abstract:**
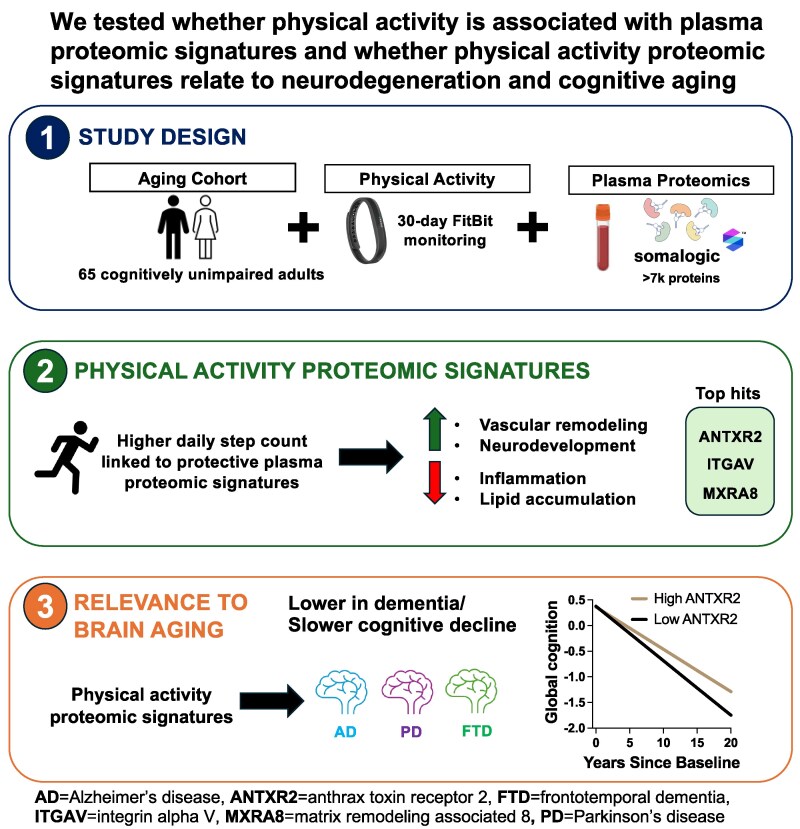

## Introduction

Dementia represents a significant global health challenge, with limited therapeutic options currently available.^1^ Physical activity (PA) is a modifiable lifestyle factor that has consistently demonstrated neuroprotective effects, including associations with lower incidence of dementia,^2^ less cortical atrophy^3,4^ and slower neurodegenerative trajectories in individuals with sporadic and even autosomal dominant dementia pathophysiology.^5-7^ Despite these promising findings, the molecular pathways underlying relationships between PA and neurodegeneration remain poorly understood. Identifying biological signatures of PA will enable stratification of individuals who may benefit most from PA interventions and could uncover novel therapeutic targets for dementia prevention.

High-throughput proteomic platforms allow for quantification of thousands of proteins in biofluid specimens to better understand the molecular drivers of human disease. These proteomic technologies, including aptamer and immunoassay-based platforms, have begun to inform novel targets for diagnosis, prognosis and risk stratification, treatment monitoring and therapeutic intervention in neurodegenerative disease.^8-11^ To date, these efforts have primarily focused on discovery of pathology-specific biomarkers and molecular pathways for discriminating symptomatic neurodegenerative disease patients from controls. By applying these sensitive molecular screening methods in cohorts with deep behavioural phenotyping and individuals that span the continuum of typical cognitive ageing to dementia, we can identify *in vivo* biological targets that underlie the neuroprotective properties of lifestyle behaviours, such as PA.

The present study leveraged six cohorts of PA and Alzheimer’s disease and related dementias (ADRD) to identify the activity-related biology that may be most relevant to dementia prevention. We first measured 7288 plasma proteins using the aptamer-based SomaScan platform in cognitively unimpaired older adults from the University of California, San Francisco (UCSF) Brain Aging Network for Cognitive Health (BrANCH) who also completed 30 days of actigraphy monitoring for objective quantification of PA. Differential regression and weighted gene co-expression network analysis (WGCNA) identified communities of plasma proteins that were robustly associated with PA. Associations of plasma proteins with PA were validated by leveraging plasma proteomic datasets from two independent cohorts with different methods of PA quantification, an endurance exercise training intervention in young to middle-aged adults^12^ and an observational study of self-reported habitual PA in racially diverse middle- to older-aged adults [Atherosclerosis Risk In Communities (ARIC) study^13^]. We next leveraged plasma proteomic, brain tissue proteomic and genomic datasets from ADRD cohorts to identify the molecular overlap between PA-related molecular pathways and ADRD risk. We identified individual plasma targets as well as broader communities of co-expressed proteins that robustly tracked with exercise, differed across symptomatic neurodegenerative conditions, prognosticated longitudinal cognitive decline and correlated with brain tissue proteomic pathways linked to cognitive resilience. These PA proteomic signatures and individual targets, which may underlie the neuroprotective effects of exercise on brain ageing, were enriched for processes linked to cell adhesion, extracellular matrix (ECM), vascular remodelling, growth factor signalling and immune response. Together, these data support the utility of leveraging exercise proteomics as a tool to probe biology relevant for dementia prevention.

## Materials and methods

### Physical activity cohorts

To evaluate the cross-cohort validity of PA plasma proteomic signatures, we included three complementary studies: (i) UCSF BrANCH to evaluate real-time objectively measured late life PA levels via actigraphy; (ii) ARIC study to evaluate self-reported PA levels in a large, diverse cohort; and (iii) Health Risk Factors, Exercise Training and Genetics (HERITAGE) to evaluate how PA proteomic signatures change following an endurance exercise intervention.

#### UCSF BrANCH

UCSF BrANCH included 65 clinically normal, community-dwelling older adults who had plasma assayed via SomaScan v4.1,^14^ which included 7288 targets across 6358 unique proteins (Supplementary Table 1), and also completed 30 days of observational Fitbit™ Flex2 PA monitoring during all waking hours. Fitbit data were quality-checked and cleaned, which included controlling for non-adherence by excluding days when there was a high suspicion that participants did not wear the device per previously published methods (i.e. days with <100 steps total).^15,16^ All BrANCH participants with plasma samples sent to SomaLogic had at least 14 days of adherence to Fitbit monitoring. Total step counts per day collected from the Fitbit device were averaged to quantify the average daily step count. BrANCH participants also underwent comprehensive neurological examinations, neuropsychological testing, study partner interview and functional assessment [Clinical Dementia Rating scale (CDR)] and blood draw. BrANCH participants were free of cognitive symptoms, free of major neurological, psychiatric or medical comorbidities (e.g. HIV, stroke, sleep apnoea), were functionally intact (CDR = 0) and were diagnosed as clinically normal per multidisciplinary consensus case conference.^17^ All participants provided written informed consent, and study procedures were approved by the UCSF Committee on Human Research.

#### ARIC

We also included publicly available published data from the ARIC cohort via Steffen *et al*., which examined plasma proteomic correlates of self-reported PA, quantified via the Baecke PA Questionnaire, in 10 644 participants from the Visit 3 examination of the ARIC study cohort [mean (SD) age: 60 (5.7) years].^13^ ARIC proteomics data were assayed on SomaScan v4.0 (∼5000 targets).

#### HERITAGE

Lastly, we included PA proteomics data from the HERITAGE cohort published by Robbins *et al*., which examined plasma protein changes before and after a 20-week endurance exercise training intervention in 654 healthy adults (ages 17–65 years).^12^ HERITAGE also included plasma proteomics pre- and post-intervention from the SomaScan v4.0 platform.

### Plasma aptamer-based proteomics

Collected venous blood was sent to SomaLogic (SomaLogic, Boulder, CO) for proteomics analysis.^14^ The SomaScan assay used in all three PA cohorts leverages short, single-stranded deoxynucleotides that bind to protein targets with high specificity.^18^ A 65 μL plasma sample was used to generate SOMAmer–protein interactions in 96-well plates. Tagged SOMAmer–protein complexes were captured using a bead-based assay, and bound SOMAmer levels were quantified via fluorescence on DNA hybridization microarrays.^14^ Signals were digitally measured as aggregated Agilent relative fluorescent units and then normalized and log2-transformed.

### PA plasma proteomic workflow

We first characterized novel associations between objectively measured PA, indexed as average daily step count and large-scale plasma proteomics in the BrANCH cohort. BrANCH also included detailed neurobehavioural assessments that enabled examination of the molecular overlap between PA and cognitive ageing outcomes. PA-related differential regression and co-expression network proteomic signatures from BrANCH were then compared with plasma proteomic signatures from the previously published HERITAGE and ARIC datasets, which leveraged other exercise quantification methods to evaluate cross-cohort consistency in the magnitude of effects and overlap of proteins significantly associated with exercise.

#### Differential regression

General linear models examined the relationship between average daily steps and plasma abundance for each protein separately, adjusting for age and sex. Model coefficients were multiplied by 1000 such that values represent the log_2_-fold protein increase/decrease associated with an increase/decrease of 1000 average daily steps. PA-related proteins were identified based on nominal statistical significance (*P* < 0.05), and top hits were further identified based on Benjamini–Hochberg false discovery rate (FDR) corrected *P* < 0.05.

#### Protein co-expression network analysis

We generated a plasma protein network (7288 log_2_ protein abundance × *n* = 65 samples) using WGCNA.^19,20^ No outliers were identified using the WGCNA sample network connectivity outlier algorithm.^19^ Network construction was performed using the blockwiseModules function using the following parameters: power = 12, deepSplit = 4, minModuleSize = 10, mergeCutHeight = 0.07, TOMdenom = ‘mean’, bicor correlation, signed network type, PAM staging and PAM respects dendro as TRUE. All samples were clustered in a single block. Module memberships were next iteratively reassigned to enforce consistency in the kME table, as previously described.^19^ Briefly, (i) proteins with intramodular kME < 0.30 were removed (unassigned grey module) and reassigned to the module with the highest kME if maximum kME > 0.30 and (ii) assigned proteins with intramodular kME > 0.10 lower than their maximum kME were reassigned to the module with their maximum kME (>0.30). Module eigengenes and signed kME values were recalculated after each reassignment, and this cleanup–recalculation process was repeated iteratively (up to 30 iterations).

#### Gene Ontology and cell-type marker enrichment

Gene Ontology (GO) enrichment for network modules and separate lists of proteins positively or negatively associated with PA (nominal *P* < 0.05) was calculated as a *P*-value (Fisher’s exact test) transformed to *Z*-score. Transformed *Z*-scores for enriched GO terms were visualized using GOparallel (https://www.github.com/edammer/GOparallel), which downloads gene sets from the Bader Lab’s monthly update.^21^ As previously published,^19^ network module enrichment for cell-type gene symbol lists was examined with one-tailed Fisher’s exact test (https://github.com/edammer/CellTypeFET). Specifically, we tested for enrichment of brain endothelial cells, pericytes, smooth muscle cells (SMC), fibroblasts, astrocytes, microglia/macrophage, neurons, oligodendrocytes and oligodendrocyte progenitor cells.^22,23^

### Cross-cohort comparisons: physical activity proteomic signatures in HERITAGE and ARIC

One-tailed Fisher’s exact tests determined BrANCH plasma network module-wise overrepresentation of proteins reported to be significantly positively or negatively associated with PA based on the HERITAGE intervention or ARIC self-reported PA. Since multivariable regression coefficients were available for all proteins measured in the ARIC study, we also correlated objectively monitored PA effect sizes (UCSF BrANCH) with self-reported PA effect sizes (ARIC) for the 4954 proteins that overlapped across BrANCH and ARIC datasets.

### PA proteomic overlap with neurobehavioural outcomes

To determine if the identified plasma PA proteomic signatures were relevant to brain health outcomes, we included detailed neurobehavioural assessments from BrANCH participants, including comprehensive neuropsychological testing and self-report questionnaires. Cognitive performance was operationalized using sample-based *Z*-score composites of episodic memory, executive functioning and processing speed, as previously described.^24^ Cognitive composites were regressed for age, sex and education based on the full sample of clinically normal BrANCH participants (*N* > 488 per composite), and residualized *Z*-scores were examined as cognitive outcomes in the present study. Processing speed *Z*-scores were reverse-coded such that higher scores translated to better (faster) performance, consistent with other cognitive outcomes. Participants also completed the 30-item self-report Geriatric Depression Scale (GDS), which quantified mood symptoms.^25,26^ GDS total scores were also regressed for demographics and reverse-coded for analysis such that higher scores translated to better (less symptomatic) mood. The *bicorAndPvalue* function from the *R* WGCNA package was used to calculate biweight midcorrelations (bicor) between average daily steps and neurobehavioural outcomes, as well as individual plasma proteins and each neurobehavioural outcome. One-tailed Fisher’s exact tests determined plasma network module-wise overrepresentation of proteins positively (protection) or negatively (risk) associated with neurobehavioural outcomes, adjusting for FDR.^12,13^

### PA proteomic overlap in symptomatic disease cohorts

We leveraged plasma proteomics data (SomaScan v4.1) from two cohorts of patients with symptomatic neurodegenerative diseases to test whether PA-related plasma protein changes in the healthy ageing BrANCH cohort overlapped with plasma protein signatures of ADRD: (i) the Stanford ADRC plasma SomaScan dataset included 120 patients with mild cognitive impairment or dementia due to Alzheimer’s disease, 113 patients with Parkinson’s disease and related disorders (i.e. Lewy body dementia) and 189 Alzheimer’s disease biomarker-negative controls; (ii) the ARTFL/LEFFTDS Longitudinal Frontotemporal Lobar Degeneration (ALLFTD) consortium (NCT04363684)^27,28^ plasma SomaScan dataset included symptomatic carriers of pathogenic mutations in frontotemporal lobar degeneration-tau [*MAPT* (*n* = 80)] or frontotemporal lobar degeneration-TDP causing genes [*C9orf72* (*n* = 149), *GRN* (*n* = 62)], patients with sporadic progressive supranuclear palsy-Richardson’s syndrome (PSP-RS; *n* = 118), which is highly specific for underlying PSP pathology,^29^ and noncarrier controls (*n* = 171) from families with a known mutation in one of the frontotemporal lobar degeneration-causing genes.^30^ Differential abundance analyses examined ADRD group differences in plasma protein abundance compared to controls. One-tailed Fisher’s exact tests examined overrepresentation of ADRD-associated proteins across BrANCH plasma network modules.

### Cognitive trajectory and brain proteome association analyses in ROSMAP

We leveraged antemortem plasma proteomic (SomaScan v4.1), longitudinal cognitive data and post-mortem brain tissue proteomic data from 436 participants in the Religious Orders Study and Rush Memory and Aging Project (ROSMAP) cohort to determine the association of PA-associated plasma proteins with cognitive trajectories and brain protein signatures of cognitive resilience. ROSMAP participants were free of dementia at study entry (baseline) and consented to clinical examinations and brain autopsy at death.^31^ The Institutional Review Board of Rush University Medical Center approved both studies. Each participant signed informed consent, the Anatomic Gift Act and a RADC Repository consent (allowing their data and biospecimens to be repurposed). Participant cognitive status at the blood draw ROSMAP timepoints used in the present analysis included 327 individuals without cognitive impairment, 81 with mild cognitive impairment and 28 with dementia. Participants completed a median [interquartile range] of 5 [3–8] cognitive assessments spanning a median [interquartile range] of 4 [2–7] years of follow-up. All participants underwent autopsy with the post-mortem dorsolateral prefrontal cortex brain tissue assayed for 8619 proteins using tandem mass tag mass spectrometry (TMT-MS) proteomics.^19^ Linear mixed-effects models examined the relationship between baseline plasma protein concentrations and longitudinal global cognitive trajectories [interaction of protein × time (years since plasma baseline)], adjusting for 10 standard neuropathological indices measured in ROSMAP: amyloid-β, tangles, cerebral amyloid angiopathy, cerebral atherosclerosis, arteriolosclerosis, Lewy body, TDP-43, gross infarct, microinfarct and hippocampal sclerosis.^19,32^ We next performed differential correlations between plasma protein levels and the 8619 brain proteins across all 436 donors, adjusting for FDR. GO enrichment of brain proteomic correlates of plasma proteins was performed as described above. To determine whether cognitive trajectory-associated plasma protein levels converged with brain proteomic signatures of cognitive resilience, we examined plasma protein correlations with brain tissue protein co-expression modules^19^ among the subset of donors with pathologically confirmed Alzheimer’s disease (*n* = 255), which included 107 brain donors who were symptomatic at death as well as 148 cognitively resilient donors who were asymptomatic at death. Results were visualized by plotting plasma protein correlation coefficients with brain tissue modules against at death versus asymptomatic at death brain module eigenprotein differences.

### Alzheimer’s disease GWAS module association

We determined whether plasma network modules linked to PA were also enriched for gene products of Alzheimer’s disease genome-wide association studies (GWAS) targets using Multi-marker Analysis of GenoMic Annotation (MAGMA; version 1.09b)^33^ and single-nucleotide polymorphism summary statistics from available Alzheimer’s disease GWAS studies,^34-36^ as previously described.^19,37^ MAGMA was performed using publicly available code: https://github.com/edammer/MAGMA.SPA. GWAS lists were filtered for genes with Alzheimer’s disease association values of *P* < 0.05 prior to MAGMA.

### Other statistics

Statistical analyses were performed in R (v4.3.1). Correlations were performed using biweight midcorrelations (bicor) or Pearson’s correlations where indicated. Two-group comparisons were examined with two-sided *t*-tests. Three or more group comparisons were examined with ANOVA with Tukey’s pairwise comparison of significance. *P* values were adjusted for multiple testing using the FDR method, where applicable.

## Results

### Fitbit proteomics cohort characteristics

An overview of all plasma proteomics cohorts and related analyses used in the present study is presented in Supplementary Table 2. Supplementary Table 3 reports detailed demographic and clinical characteristics for the 65 cognitively unimpaired older adults (CDR = 0) who completed Fitbit^TM^ monitoring and plasma proteomics through the UCSF BrANCH study. Participants were on average 76.7 years old (range = 59–91) with 17.6 years of education, 60% female, and a majority identified as non-Hispanic White (88%). Average daily step counts ranged from 669 to nearly 13 845 steps per day, with a sample mean of 7277 steps per day. Collectively, the distribution of demographic factors and step count for the BrANCH subcohort with plasma proteomics closely mirrored the characteristics of the larger BrANCH cohort (*n* = 175) that underwent Fitbit^TM^ monitoring (mean age: 73.7 years, 17.6 years of education, 58% female, 86% non-Hispanic White, 6964 average daily step count).^16^

### Network analysis highlights ECM and lipid homeostasis signatures of physical activity

**Figure 1:**
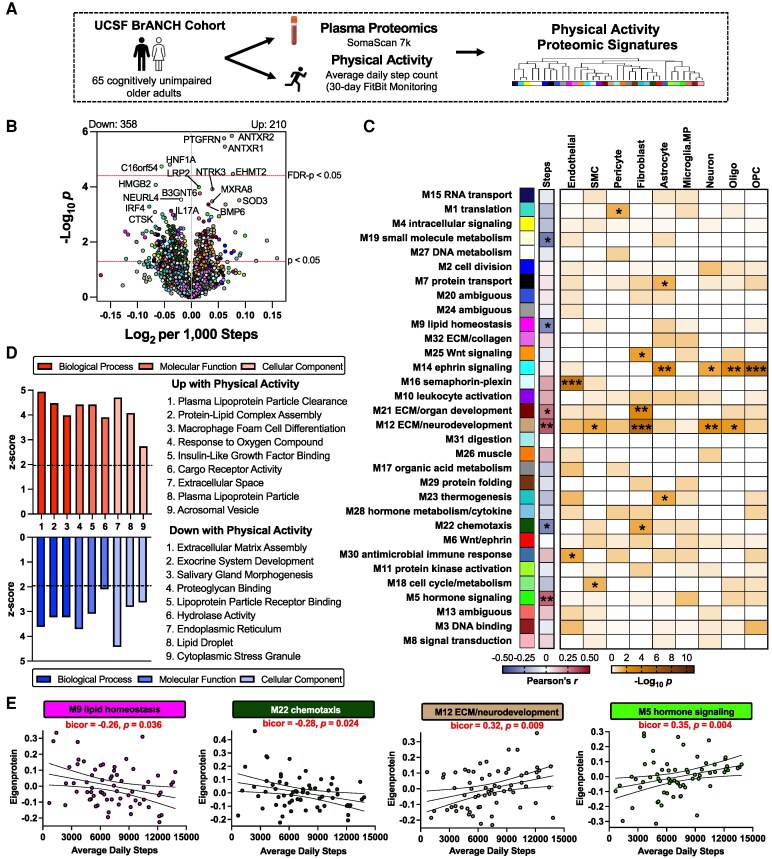
Plasma proteomic signatures of PA. (**A**) Plasma samples from 65 cognitively unimpaired adults in the UCSF Brain Aging Network for Cognitive Health (BrANCH) were analysed using large-scale aptamer-based proteomics [SomaScan v4.1 (7k proteins)]. Participants also completed 30-day Fitbit monitoring for quantification of PA, operationalized as average daily step count, which was integrated with proteomic data to identify plasma proteomic signatures of PA. (**B**) Differential regression models examined correlations between average daily step count and plasma protein abundance, adjusting for age and sex (see Supplementary Table 1 for protein annotations and Supplementary Table 4). Volcano plot depicts the log_2_-fold change in protein abundance for a 1000-step increase in average daily step count by negative log_10_  *P*-value from multiple linear regression models. Proteins are coloured by the co-expression module they were assigned according to weighted gene co-expression network analysis (WGCNA). (**C**) GO analysis identified biological pathways associated with proteins that increased or decreased with higher PA (see Supplementary Table 5). Enrichment for a given ontology is shown by *Z*-score, transformed from a Fisher’s exact test. (**D**) WGCNA identified 32 plasma protein co-expression modules (see Supplementary Table 6). GO analysis was used to identify the principal biology represented by each module (see Supplementary Table 7), and module relatedness is shown in the dendrogram to the right. Heatmaps represent module eigenprotein correlations with PA (*N* = 65; see Supplementary Table 8) and module-wise cell-type enrichment, assessed by module protein overlap (Fisher’s exact test) with nine cell-type-specific marker lists: brain endothelial cells, pericytes, SMC, fibroblasts, astrocytes, microglia/macrophages, neurons, oligodendrocytes, and oligodendrocyte progenitor cells. ***P < 0.001, **P  *<* 0.01, *P < 0.05. (**E**) Scatterplots of PA correlated against select plasma protein co-expression module eigenproteins [datapoints represent individual subjects (*N* = 65)].

We performed age- and sex-adjusted differential regressions to identify individual plasma proteins that were positively or negatively associated with PA (Supplementary Table 4). Roughly 8% of the plasma proteome was nominally associated with PA (unadjusted *P* < 0.05), with 210 proteins positively associated with step count and 358 proteins negatively associated with step count (Fig. 1A and B). Six proteins survived FDR-correction, including higher abundance of anthrax toxin receptor cell adhesion molecules 1 and 2 (ANTXR1, ANTXR2; aka, capillary morphogenesis gene 1 and 2, respectively) as the top two PA hits by statistical significance. GO analysis of PA-associated proteins (Fig. 1C; Supplementary Table 5) revealed strong links to lipoprotein and ECM biology, including plasma lipoprotein particle clearance and cell adhesion (increased with PA), as well as lipoprotein particle receptor binding and ECM assembly (decreased with PA).

We next leveraged WGCNA to identify communities of plasma proteins that were co-expressed across plasma samples and associated with PA. WGCNA revealed 32 protein co-expression modules (Fig. 1D). Protein module membership assignments are provided in Supplementary Table 6. Pathway and cell-type enrichment analyses were performed to identify the primary ontology used for module annotation (Supplementary Table 7; Supplementary Fig. 1). Nine modules exhibited significant overrepresentation for individual PA-associated proteins identified in differential regression (FDR-*P* < 0.05), and of those nine, six had module eigenproteins that were significantly correlated with PA (Supplementary Table 8). Module 12 (M12) ECM/neurodevelopment, comprised of 165 proteins, was enriched for ontology terms related to neurodevelopment and ECM/cell adhesion, as well as vascular (fibroblast, SMC), neuronal and oligodendrocyte cell-type markers. M12 module eigenprotein levels positively correlated with PA (bicor = 0.32, *P* = 0.009; Fig. 1E), and M12 also harboured the highest proportion of individual proteins that were positively associated with PA [54 out of 165 proteins (33%)], including four proteins that reached FDR significance from differential regression [ANTXR1, ANTXR2, prostaglandin F2 receptor inhibitor (PTGFRN), euchromatic histone-lysine N-methyltransferase 2 (EHMT2)]. PA was also positively correlated with M21 ECM/organ development (bicor = 0.25, *P* = 0.046), also enriched for fibroblast markers and closely related to M12, and M5 hormone signalling (bicor = 0.35, *P* = 0.004). M19 small molecule metabolism (bicor = −0.30, *P* = 0.015), M9 lipid homeostasis (bicor = −0.26, *P* = 0.036) and M22 chemotaxis (bicor = −0.28, *P* = 0.024) exhibited the strongest negative associations with PA.

### Physical activity proteomic signatures replicate across-cohorts and methods of physical activity quantification

**Figure 2:**
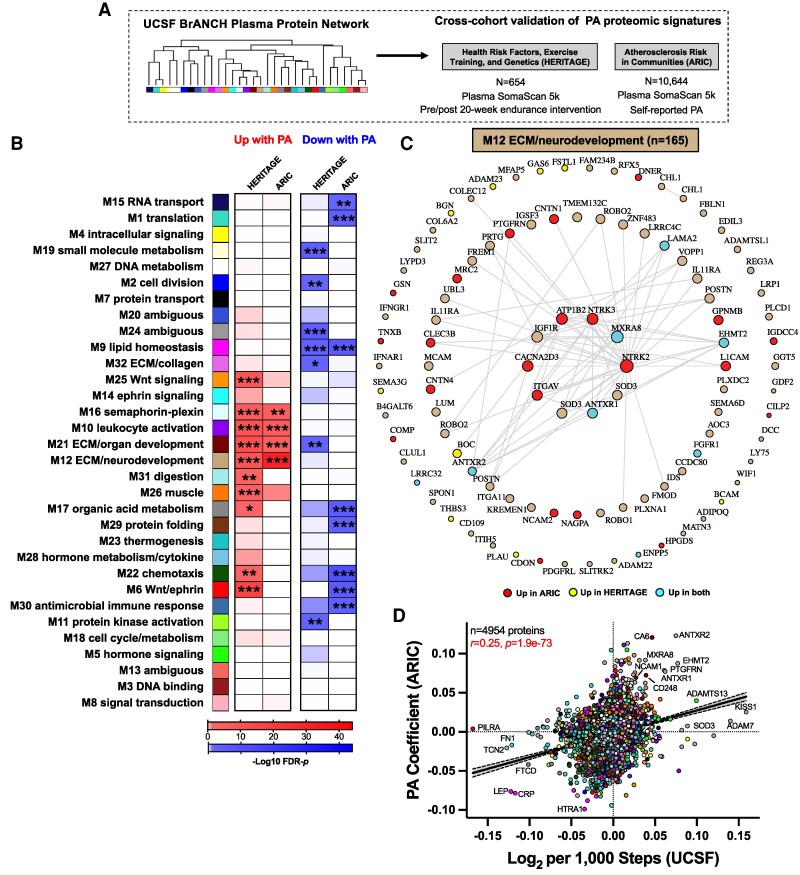
PA plasma proteomic signatures are conserved across-cohorts and methods of PA quantification. (**A**) BrANCH plasma network module proteins were cross-referenced with summary statistics from two independent studies that examined plasma proteomic [SomaScan v4.0 (5k)] correlates of an endurance training intervention [Health, Risk Factors, Exercise, Training and Genetics (HERITAGE) study; Robbins *et al*.^12^] and self-reported PA [Atherosclerosis Risk in Communities (ARIC) study; Steffen *et al*.^13^]. (**B**) BrANCH plasma network module (*N* = 32 modules) protein overlap with proteins that changed pre-/post-HERITAGE intervention (*N* = 5578 overlapping protein measurements)^12^ or correlated with self-reported PA in ARIC (*N* = 4954 overlapping protein measurements).^13^ One-tailed Fisher’s exact test was used to determine module-wise enrichment, and results were FDR-corrected using the Benjamini–Hochberg method (see Supplementary Table 9). ***P < 0.001, **P  *<* 0.01, *P < 0.05. (**C**) Top 100 proteins by module connectivity (i.e. correlation with module eigenprotein value) for the M12 ECM/neurodevelopment module (see Supplementary Table 1 for protein annotations). Larger circles represent larger correlations or module ‘hub’ proteins. Proteins that increased in HERITAGE, ARIC or both are highlighted. (**D**) Fitbit-based PA proteomic effect sizes in BrANCH were correlated (Pearson’s) against self-reported PA proteomic effect sizes in ARIC [datapoints represent individual proteins (*N* = 4954 overlapping protein measurements)].

The plasma proteomic signatures of PA identified in the BrANCH cohort were based on observational actigraphy monitoring of PA in older adults. To determine whether these PA plasma proteomic signatures were robust to cohort composition and method of PA quantification, we cross-referenced summary statistics from two independent studies that examined plasma SomaScan proteins and PA (Fig. 2A): (i) the young to middle-aged HERITAGE exercise trial (*N* = 654) with plasma proteins measured pre- and post-endurance training intervention^12^ and (ii) middle to old-aged ARIC cohort (*N* = 10 644) with plasma proteins correlated against self-reported habitual PA.^13^ Most modules linked to PA in the Fitbit-based BrANCH cohort were enriched for proteins linked to PA in the two independent datasets (FDR-*P* < 0.05; Supplementary Table 9), with strong concordance in directionality (Fig. 2B). Specifically, M12 and M21 were enriched for proteins that increased post-intervention in HERITAGE and proteins positively correlated with self-reported PA in ARIC (FDR-*P* < 0.05). PA-associated proteins from HERITAGE and ARIC that overlap with M12 module proteins by intramodular connectivity are shown in Fig. 2C. M17 and M9 were enriched for proteins that decreased post-intervention in HERITAGE and, alongside M22, proteins negatively correlated with self-reported PA in ARIC (FDR-*P* < 0.05).

Correlation of Fitbit-based coefficients in BrANCH to self-reported PA-based coefficients in ARIC (*N* = 4954 overlapping protein measurements) also supported the concordant directionality of PA plasma proteomic signatures (*r* = 0.25, *P* = 1.9e-73; Fig. 2D). ANTXR2, the top hit in BrANCH, was also the top hit in ARIC in terms of statistical significance. In addition to ANTXR2, other hub proteins from M12 [matrix remodelling-associated protein 8 (MXRA8), EHMT2, ANTXR1, PTGFRN, neurotrophic tyrosine kinase receptor type 3] and M21 [CD248, neural cell adhesion molecule 1 (NCAM1)] exhibited strong associations with PA in ARIC.

### Physical activity proteomic signatures converge with neurobehavioural outcomes

**Figure 3:**
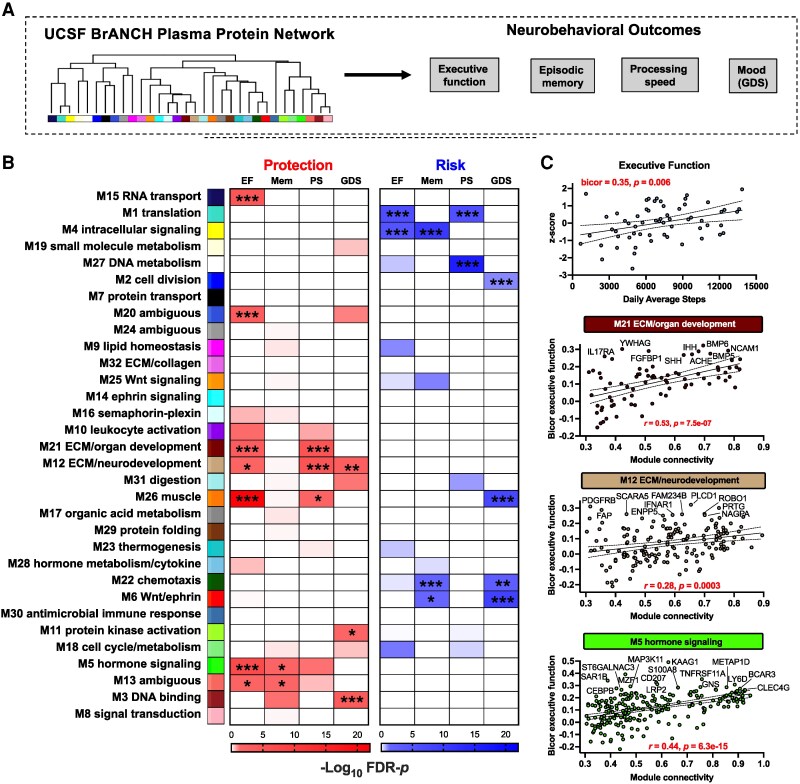
PA-related plasma proteins predict age-related neurobehaviour. (**A**) Plasma network module proteins were correlated against neurobehavioural outcomes measured in BrANCH [*N* = 65; executive function, episodic memory, processing speed and mood (GDS)]. (**B**) BrANCH plasma network module (*N* = 32 modules) protein overlaps with proteins positively (protection) or negatively (risk) correlated with neurobehaviour. One-tailed Fisher’s exact test was used to determine module-wise enrichment, and results were FDR-corrected using the Benjamini–Hochberg method (see Supplementary Table 10). ***P < 0.001, **P  *<* 0.01, *P < 0.05. (**C**) Scatterplots display the correlation (bicor) between PA and executive function [datapoints represent individual subjects (*N* = 65)] as well as the correlation (Pearson’s) between an individual protein’s strength of connectivity to a module and the individual protein’s association with executive function [datapoints represent individual proteins (M21: *N* = 78 proteins; M12: *N* = 165 proteins; M5: *N* = 230 proteins)]. Annotated proteins (Supplementary Table 1) are module members that were significantly correlated with executive function (bicor *P* < 0.05).

To understand the relevance of identified PA proteomic signatures to cognitive ageing, we next correlated plasma proteins with neurobehavioural outcomes (cognitive composites, mood) in BrANCH and tested for module-wise overrepresentation of neurobehavioural-associated proteins (Fig. 3A). All three modules that were positively associated with PA (M21, M12, M5) also harboured proteins that were positively associated with neurobehaviour (Fig. 3B; Supplementary Table 10): executive function (M21, M12, M5), memory (M5), processing speed (M21, M12) and depressive symptoms (M12). To determine whether individual proteins linked to neurobehaviour were also of high influence (‘hub proteins’) in M21, M12 and M5, we plotted individual protein correlational effect sizes against their intramodular connectivity; we focused on executive function given its relationship to PA (bicor = 0.35, *P* = 0.006) and all three modules. For all three modules, proteins with higher intramodular connectivity exhibited larger correlations with executive function (Fig. 3C), including M21 and M12 proteins linked to growth factor signalling/neurodevelopment [M21: NCAM1, bone morphogenetic proteins 5 and 6 (BMP5, BMP6); M12: protogenin, roundabout guidance receptor 1 (ROBO1)] and M5 proteins involved in immune response [C-type lectin domain family 4 member G (CLEC4G), TNF receptor superfamily member 11a (TNFRSF11A)]. Of the three modules that were negatively associated with PA, only M22 chemotaxis exhibited overrepresentation of proteins negatively correlated with neurobehaviour, specifically memory and depressive symptoms. Taken together, these results demonstrate molecular overlap between PA and age-related neurobehaviour, reflected by shared plasma proteomic signatures linked to neurodevelopment and immune signalling.

### M12 ECM/neurodevelopment proteins are decreased across multiple neurodegenerative diseases

**Figure 4:**
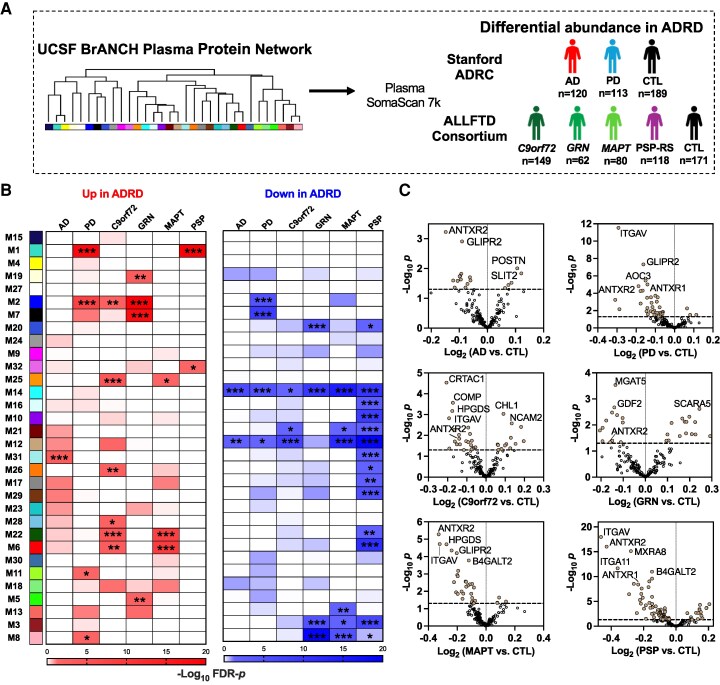
PA plasma proteomic signatures are altered across Alzheimer’s disease and related dementias. (**A**) BrANCH plasma network module proteins were cross-referenced with differentially abundant plasma proteins in symptomatic Alzheimer’s disease and related dementias (ADRD). ADRD plasma proteomics data [SomaScan v4.1 (7k)] were sourced from two independent datasets, the Stanford ADRC (Alzheimer’s disease, Parkinson’s disease, control) and the ALLFTD consortium [genetic (*C9orf72*, *GRN*, *MAPT*) and sporadic frontotemporal lobar degeneration (PSP-RS), controls]. (**B**) BrANCH plasma network module (*N* = 32 modules) protein overlap with proteins that increased or decreased in symptomatic ADRD. One-tailed Fisher’s exact test was used to determine module-wise enrichment, and results were FDR-corrected using the Benjamini–Hochberg method (see Supplementary Table 11). ***P < 0.001, **P  *<* 0.01, *P < 0.05. (**C**) Volcano plots display differential abundance of M12 ECM/neurodevelopment proteins (*N* = 165 proteins) in each ADRD group (statistical significance defined by nominal ANOVA *P* < 0.05). See Supplementary Table 1 for protein annotations.

After establishing the cross-cohort reproducibility of PA plasma proteomic signatures and their convergence with neurobehavioural ageing in cognitively unimpaired older adults, we next asked whether alterations in PA plasma proteomic signatures could be detected in symptomatic patients with ADRD. Thus, we leveraged plasma SomaScan datasets from the Stanford ADRC, which included patients with symptomatic Alzheimer’s disease and Parkinson’s disease and the ALLFTD consortium, which included patients with symptomatic familial or sporadic frontotemporal lobar degeneration spectrum disorders (Fig. 4A). Module overrepresentation analysis indicated that M12 was enriched for plasma proteins with lower abundance across most ADRD groups compared to controls (FDR-*P* < 0.05; Fig. 4B; Supplementary Table 11). Similarly, M21 exhibited a pattern of enrichment for proteins with lower abundance in *C9orf72*, *MAPT* and sporadic PSP-RS.

We next more deeply investigated individual proteins from M12, given the strong pattern of findings demonstrating its enrichment for plasma proteins that were reliably higher with PA in healthy adults and reliably lower across ADRD groups. First, we examined volcano plots of differential abundance of M12 proteins in each ADRD group (versus controls) to identify individual proteins driving the observed patterns (Fig. 4C). ANTXR2 and integrin subunit alpha V (ITGAV), another M12 hub protein related to PA, both exhibited lower abundance across all six ADRD conditions compared to controls. ANTXR2 was the top M12 hit in Alzheimer’s disease and *MAPT* mutation carriers, and ITGAV was the top hit in Parkinson’s disease and PSP-RS. In addition to ANTXR2 and ITGAV, seven other ECM and cell adhesion-related proteins from M12 that positively related to PA in prior analysis also exhibited lower abundance in over half of the ADRD groups examined (i.e. 4 or more): ANTXR1, integrin subunit alpha 11, matrix remodelling-associated 8 (MXRA8), GLI pathogenesis-related 2 (GLIPR2), alpha-1,6-mannosylglycoprotein 6-beta-N-acetylglucosaminyltransferase, C-type lectin domain family 3 member B (CLEC3B) and haematopoietic prostaglandin D synthase (HPGDS).

### Plasma ANTXR2 predicts cognitive decline and Alzheimer’s disease brain proteomic signatures of cognitive resilience

**Figure 5:**
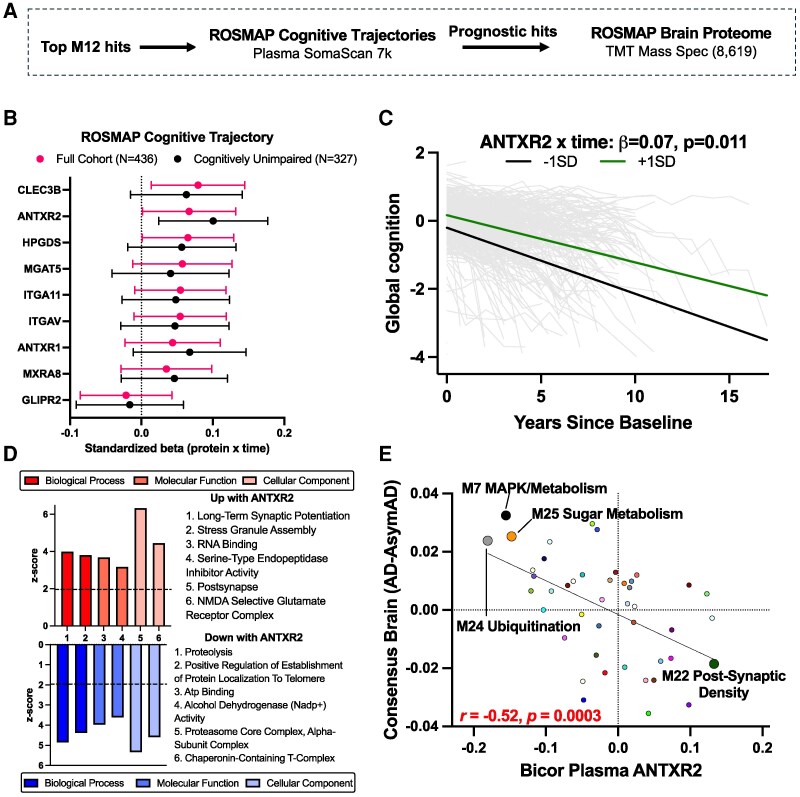
Plasma ANTXR2 predicts cognitive trajectories and brain tissue protein signatures in ROSMAP. (**A**) Plasma proteomics [SomaScan v4.1 (7k)] were measured at baseline in 436 ROSMAP participants who completed antemortem longitudinal cognitive testing and post-mortem dorsolateral prefrontal cortex brain tissue proteomics [tandem mass tag mass spectrometry (TMT-MS) proteomics^19^]. Top plasma hits from M12 were entered as predictors of global cognitive trajectories and brain tissue protein abundance. (**B**) Forest plot displays effect sizes of M12 proteins on global cognitive trajectories from linear mixed-effects models with subject-specific random intercepts and slopes in the full cohort and the subset without cognitive impairment at baseline (*N* = 327). Points represent the standardized beta of protein × time on cognitive trajectories, and whiskers represent 95% confidence intervals. (**C**) Spaghetti plot displays model-estimated global cognitive trajectories at high (1SD) and low levels (−1SD) of plasma ANTXR2 overlaid on subject-specific raw data across the full cohort. (**D**) Bar plots display results of GO analysis for post-mortem brain tissue proteins associated with higher or lower antemortem plasma abundance of ANTXR2. Enrichment for a given ontology is shown by *Z*-score, transformed from a Fisher’s exact test. (**E**) Scatterplot displays the correlation (Pearson’s) between plasma ANTXR2 correlations with ROSMAP brain tissue network modules and brain tissue modules eigenprotein differences for symptomatic versus asymptomatic Alzheimer’s disease (as reported by Johnson *et al*.^19^). Brain tissue modules that differed between symptomatic and asymptomatic Alzheimer’s disease and significantly correlated with ANTXR2 (see Supplementary Table 12) are annotated and displayed with larger circles [datapoints represent individual modules (*N* = 44 modules)].

We identified nine key M12 proteins that positively related to PA and were reliably lower in the symptomatic stage of multiple ADRDs (≥4); however, it remained unclear whether these plasma protein signals in symptomatic patients could predict future cognitive decline and reflected brain-based pathophysiological processes. To address these questions, we leveraged plasma proteomic (SomaScan) and brain tissue proteomic (TMT-MS^19^) data from 436 ROSMAP participants (baseline diagnoses: *N* = 327 cognitively unimpaired, *N* = 80 mild cognitive impairment, *N* = 28 dementia) who completed antemortem blood draws and longitudinal cognitive assessments prior to brain donation (Fig. 5A). Cognitive analyses were first performed in the full sample and next in the subset of participants who were cognitively unimpaired at the time of blood draw, allowing us to test the prognostic value of PA-associated M12 hits measured during the presymptomatic phase of disease. Adjusting for ten neuropathologic indices, higher antemortem levels of ANTXR2, HPGDS and CLEC3B significantly predicted slower global cognitive decline (Fig. 5B). In the subset of individuals who were cognitively unimpaired at baseline, only higher levels of ANTXR2 significantly predicted slower global cognitive decline (Fig. 5C).

We next leveraged the ROSMAP TMT-MS brain dataset^19^ to identify brain proteomic pathways associated with peripheral abundance of ANTXR2. Differential correlation across 8619 TMT-MS brain proteins (Supplementary Table 12) revealed enrichment of synaptic signalling pathways with higher plasma abundance of ANTXR2 (e.g. ‘long-term synaptic potentiation’) and enrichment of proteolytic pathways with lower plasma abundance of ANTXR2 (e.g. ‘proteolysis’; Fig. 5D). Notably, plasma ANTXR2 did not correlate with brain TMT-MS measurement of ANTXR2 (bicor*<*0.01, *P* = 0.962). To further inform brain-based pathways of cognitive resilience that would be relevant for dementia prevention, we examined correlations of plasma ANTXR2 with brain protein co-expression modules in the subset of cases with autopsy-confirmed Alzheimer’s disease (107 symptomatic Alzheimer’s disease, 148 asymptomatic Alzheimer’s disease). ANTXR2 was significantly correlated with five previously identified brain protein co-expression modules.^19^ ANTXR2 positively correlated with brain M22 post-synaptic density, which had higher abundance in asymptomatic Alzheimer’s disease versus symptomatic Alzheimer’s disease, and negatively correlated with brain M7 MAPK/metabolism, brain M24 Ubiquitination and brain M25 Sugar Metabolism, which had lower abundance in asymptomatic Alzheimer’s disease versus symptomatic Alzheimer’s disease (Fig. 5E).

Collectively, these results indicate that blood-based ANTXR2 can forecast preclinical cognitive decline and converge with synaptic and proteolytic brain proteomic signatures of cognitive resilience. These findings may reflect the unique contributions of peripherally derived ANTXR2, our top Fitbit-based hit for PA, given the poor correlation of ANTXR2 between blood and the brain.

### M12 ECM/neurodevelopment is enriched for Alzheimer’s disease genetic risk loci

**Figure 6:**
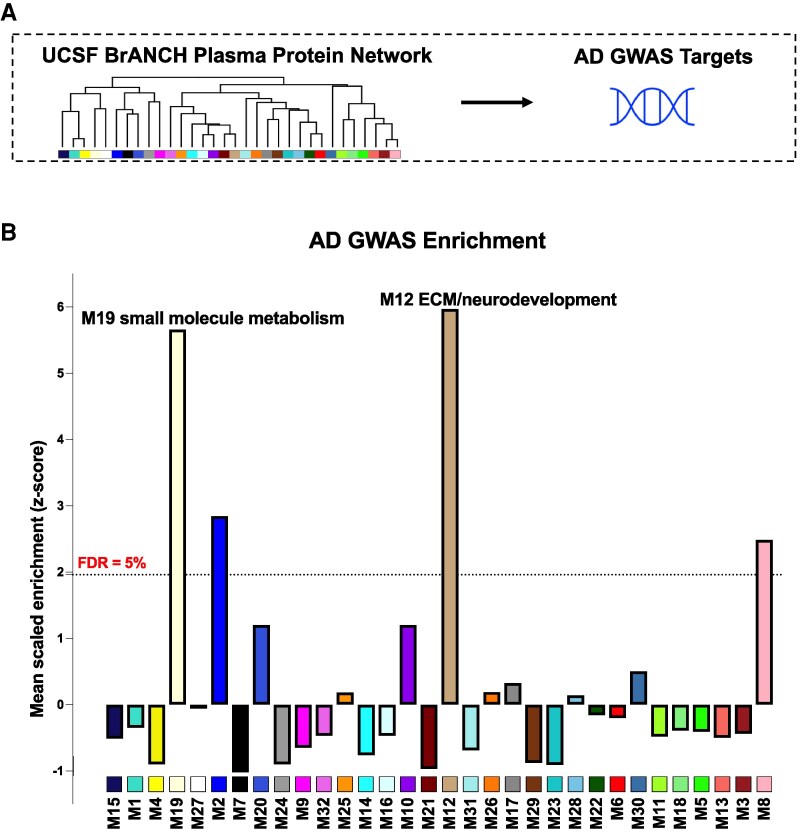
M12 ECM/neurodevelopment is enriched for Alzheimer’s disease genetic risk loci. (**A**) BrANCH plasma network modules (*N* = 32 modules) were tested for enrichment of proteins from Alzheimer’s disease genetic risk loci (*N* = 4476 genes) using Multi-marker Analysis of GenoMic Annotation (MAGMA) based on summary statistics from available Alzheimer’s disease genome-wide association studies (GWAS).^34-36^ (**B**) The dashed line indicates a mean scaled enrichment *Z*-score of 1.96 [false discovery rate (FDR)-*P* = 0.05], above which enrichment was considered significant (see Supplementary Table 13). Modules are ordered by relatedness as illustrated in Fig. 1D.

As an environmental factor, PA could in part influence disease pathophysiology by interacting with molecular pathways that are also regulated by genetic risk factors. To interrogate the molecular overlap between PA and genetic dementia risk, we tested PA-related plasma modules for enrichment of GWAS hits using MAGMA (Supplementary Table 13). We specifically focused on Alzheimer’s disease GWAS targets given the large power of the extant Alzheimer’s disease GWAS literature^35^ and thus higher confidence in Alzheimer’s disease GWAS hits, as well as our findings linking PA plasma signatures to the ROSMAP Alzheimer’s disease brain proteome. Four plasma modules were significantly enriched for Alzheimer’s disease GWAS targets [MAGMA (FDR) *P* < 0.05], including PA-related modules M12 ECM/neurodevelopment and M19 small molecule metabolism (Fig. 6). M12 featured 41 gene products from candidate Alzheimer’s disease genes, including top PA hits ANTXR1 and EHMT2. GO analysis of these 41 Alzheimer’s disease risk loci found in M12 revealed ontological enrichment for ‘heparin-binding’ proteins, specifically apolipoprotein A5, CLEC3B, follistatin like 1, slit guidance ligand 3 and superoxide dismutase 3. M19, which negatively correlated with PA, featured 21 gene products from candidate Alzheimer’s disease genes. The MAGMA enrichment of M19 was strongly driven by APOE (-log_10_ GWAS *P* = 72.7), also a heparin-binding protein; of note, all 4 SOMAmer targets for APOE protein resided in M19. GO analysis did not reveal a clear ontological signal for these 21 Alzheimer’s disease risk loci in M19. Taken together, integration of Alzheimer’s disease GWAS data with our plasma protein network again showcased M12 as harbouring multiple PA-associated targets with potential causative links to Alzheimer’s disease, possibly via heparin-binding processes known to influence amyloid fibril formation.^38,39^

## Discussion

This study integrated large-scale plasma proteomics with actigraphy-based step count data to identify molecular signatures of PA in ageing adults. Our systems biology approach highlighted ECM biology related to cell adhesion and angiogenesis, growth factor signalling, lipid homeostasis and immuno-inflammatory biology as key pathways associated with objective PA levels. These pathways and individual proteins from PA-related co-expression modules were robustly associated with PA in two independent exercise cohorts, supporting the rigour and reproducibility of these exercise-related targets. In additional independent cohorts, the most concordant exercise-related proteins and pathways also differed by Alzheimer’s disease /ADRD diagnosis, predicted longitudinal cognitive trajectories and brain tissue proteomic signatures of cognitive resilience, and exhibited overlap with Alzheimer’s disease genetic risk loci, thereby highlighting a convergent biological link between blood-based markers of PA and neurodegenerative disease pathophysiology. ANTXR2, an ECM/cell adhesion molecule involved in angiogenesis and vascular remodelling,^40^ showed some of the strongest relationships with both PA- and ADRD-related clinical and biological outcomes. Taken together, these findings (i) highlight molecules involved in ECM, lipid and immunovascular pathways as potential exercise-associated Alzheimer’s disease/ADRD prevention targets, (ii) advance the field towards biologically informed implementation of lifestyle intervention for precision dementia prevention and (iii) integrate multiple bioinformatic techniques to develop a resource of exercise-related biology that warrants additional in-depth investigation.

The top exercise modules (M12 and M21) and individual proteins (e.g. ANTXR2/1, MXRA8, ITGAV) suggest the key role of activity-associated increases in peripheral ECM, particularly fibroblast activation and vascular remodelling, that associate with cognitive trajectories and are subsequently decreased across Alzheimer’s disease /ADRDs. These findings broadly align with the extant body of research linking PA to vascular health and further refine potential targets to changes in the structure and plasticity of the vascular matrix.^41^ Vascular ECM is comprised of a network of molecules that provide crucial structural and functional support for blood vessels, including maintaining integrity, elasticity, angiogenic repair/remodelling and regulation of blood flow^42^; a key target of exercise may therefore function through remodelling and rejuvenation of vascular molecules. These findings also align with an increasing number of proteomic studies in Alzheimer’s disease that are converging on both peripheral and brain ECM pathways as disease hubs. For instance, in autosomal dominant and sporadic Alzheimer’s disease, proteins in CSF and brain tissue reflecting ECM processes (e.g. SPARC-related modular calcium binding 1, spondin 1) are consistently identified as top hits differentiating controls from Alzheimer’s disease, evidence dysregulation decades before symptom onset, and associate with early formation of amyloid plaques.^19,43,44^ Using unbiased plasma proteomics, our work further extends these findings to suggest a role for the *peripheral* ECM for brain health and highlights the ECM as a potential therapy target underlying how exercise may support dementia prevention in humans.

Furthermore, within the M12 ECM/neurodevelopment module, ANTXR2, MXRA8 and ITGAV were among the top individual hits most strongly increased with PA and decreased across ADRD syndromes. ANTXR2, aka capillary morphogenic gene 2 (CMG2), was canonically discovered as a receptor for the anthrax toxin virus but was subsequently found to have key roles in normal physiology, including repair and stabilization of blood vessels, ECM organization and cell adhesion to ECM and vascular tissue remodelling.^45,46^ Similarly, MXRA8 is a cell surface protein also originally identified for its role as a receptor facilitating entry of arthritogenic alphaviruses.^47^ MXRA8 is strongly expressed in joint and skeletal muscle tissue and involved in response to tissue injury,^48^ raising the possibility that MXRA8 possesses ‘myokine’-like properties with release from skeletal/muscle tissue following exercise-related contractions. Integrins are heterodimeric membrane proteins composed of alpha and beta subunits that facilitate cell-ECM interactions. In the present study, the aptamer for ITGAV that demonstrates the strongest associations with PA and ADRDs targeted the ITGAV:ITGB3 heterodimer (Somalogic sequence id: 20187-10), which functions as a receptor for numerous ECM-interacting proteins involved in angiogenesis (e.g. fibronectin) and haemostasis (e.g. vitronectin).^49^ Together, these targets support the notion that exercise supports tissue-related plasticity that is associated with neuroprotective outcomes. It is unclear if these proteins cross the blood–brain barrier, and in fact, we did not observe a strong association between peripheral and brain tissue levels of ANTXR2; however, ANTXR2 co-expressed with neuronal and oligodendrocyte markers in the plasma network module M12 and exhibited significant associations with brain tissue proteins reflecting synaptic signalling and proteolytic functions, suggesting downstream neuroprotective associations in the CNS. Nonetheless, given the primarily observational nature of our data, future work is needed to determine the tissue and cellular origin and mechanistic importance of promising exercise molecules in non-human Alzheimer’s disease /ADRD models.

Other top exercise pathways that overlapped with brain ageing outcomes in our study also highlighted molecules involved in bone and growth factor signalling (e.g. BMP5, BMP6, CLEC3B) as well as immune response (e.g. CLEC4G, TNFRSF11A). Immune benefits of PA are extensively documented.^50^ These data suggest additional specificity such that bone-associated immune signalling may link PA and cognitive outcomes. Indeed, emerging data support a bone–brain axis in which communication between bone-derived molecules may drive brain outcomes.^51^ In the CNS, PA is strongly linked to decreased microglial and astrocytic activation in animal models,^52-54^ and emerging data support the relevance of these exercise immune pathways in humans.^55^ Deeper investigation of how the immune system may serve as a communication axis between peripheral organ systems, including the bone, to the brain following exercise is likely high yield avenues of future work.

Our study has several limitations. The BrANCH cohort with objectively monitored PA was modest in sample size and reflects naturalistic, observational monitoring rather than a randomized intervention, introducing the potential for residual confounding (e.g. healthier individuals engaging in greater activity). However, we incorporated proteomic data from external cohorts with orthogonal methods of PA quantification, including a pre-/post-exercise intervention (HERITAGE), to validate the relevance of targets and pathways most strongly associated with PA in BrANCH. We exclusively leveraged SomaScan datasets in cross-cohort analyses to maintain consistency in plasma proteomic coverage; however, this approach limited our blood-based biological discovery and validation to targets measured on SomaScan, which may introduce bias in the proteomic pathways identified. Nonetheless, we showed that candidate plasma SomaScan hits in ROSMAP meaningfully associated with brain tissue proteomics assayed via an orthogonal technique, TMT-MS, supporting the potential generalizability and robustness of our findings. Another limitation is that the BrANCH cohort primarily consisted of highly educated white individuals. Despite this, our results showed consistency across a more diverse ageing cohort in the ARIC study. Importantly, with the exception of the HERITAGE exercise trial, most of the data incorporated in this study were observational. The directionality of the top exercise pathways and individual targets on ADRD-related outcomes therefore cannot be currently determined, though our inclusion of genetic and longitudinal cognitive data helps mitigate this concern. Future research with mechanistic models is needed to explore the potential of candidate exercise markers as therapeutic targets for dementia prevention.

Identifying the biology of well-known resilience behaviours such as PA can be used to unlock new insights into dementia prevention and treatment. Here we provide an in-depth interrogation of peripheral exercise signatures for future studies to build upon. Further examination and experimental modulation of the identified proteins, including those represented in M12 ECM/neurodevelopment, are next steps towards validating these as potential biomarkers and therapeutic targets. With an eye towards precision medicine, our findings also support the use of plasma proteomics to characterize individual biological signatures and identify people at greatest risk of adverse brain ageing who may benefit most from exercise intervention.

## Supplementary Material

fcag287_Supplementary_Data

## Data Availability

UCSF BrANCH data are available on request made to the UCSF Memory and Aging Center. Academic; not-for-profit investigators can request data for professional education and for research studies. Requests can be made online (https://memory.ucsf.edu/research-trials/professional/open-science). Datasets used for the analyses for the current study are also available from the corresponding author on reasonable request. Algorithms used for protein data processing and analysis are available in existing R packages and at https://github.com/edammer, as described in the Methods. ROSMAP resources can be requested at https://www.radc.rush.edu and www.synapse.org. Pre-existing data access policies for each of the ARIC parent cohort studies specify that research data requests can be submitted to each steering committee; these will be promptly reviewed for confidentiality or intellectual property restrictions and will not unreasonably be refused. Please refer to the data sharing policies of these studies. Individual-level patient or protein data may further be restricted by consent, confidentiality or privacy laws/considerations. These policies apply to both clinical and proteomic data.
