# Supplementary material for: Plasma proteomics reveals molecular overlap between physical activity and dementia risk
Source: Brain Commun. 2026 Jul 21;8(4):fcag287. doi: 10.1093/braincomms/fcag287 (PMC13426312; doi:10.1093/braincomms/fcag287)
Supplement: fcag287_Supplementary_Data [file fcag287_supplementary_data.zip › Supplementary_Figure_1.pdf]

Supplementary Figure 1. **Pathway Analysis on Plasma Protein Network Modules.** Gene ontology (GO) analysis was performed to ascertain the principal biology represented by the constituent proteins in each module. Enrichment for a given ontology is shown by z score, transformed from a Fisher's exact test.

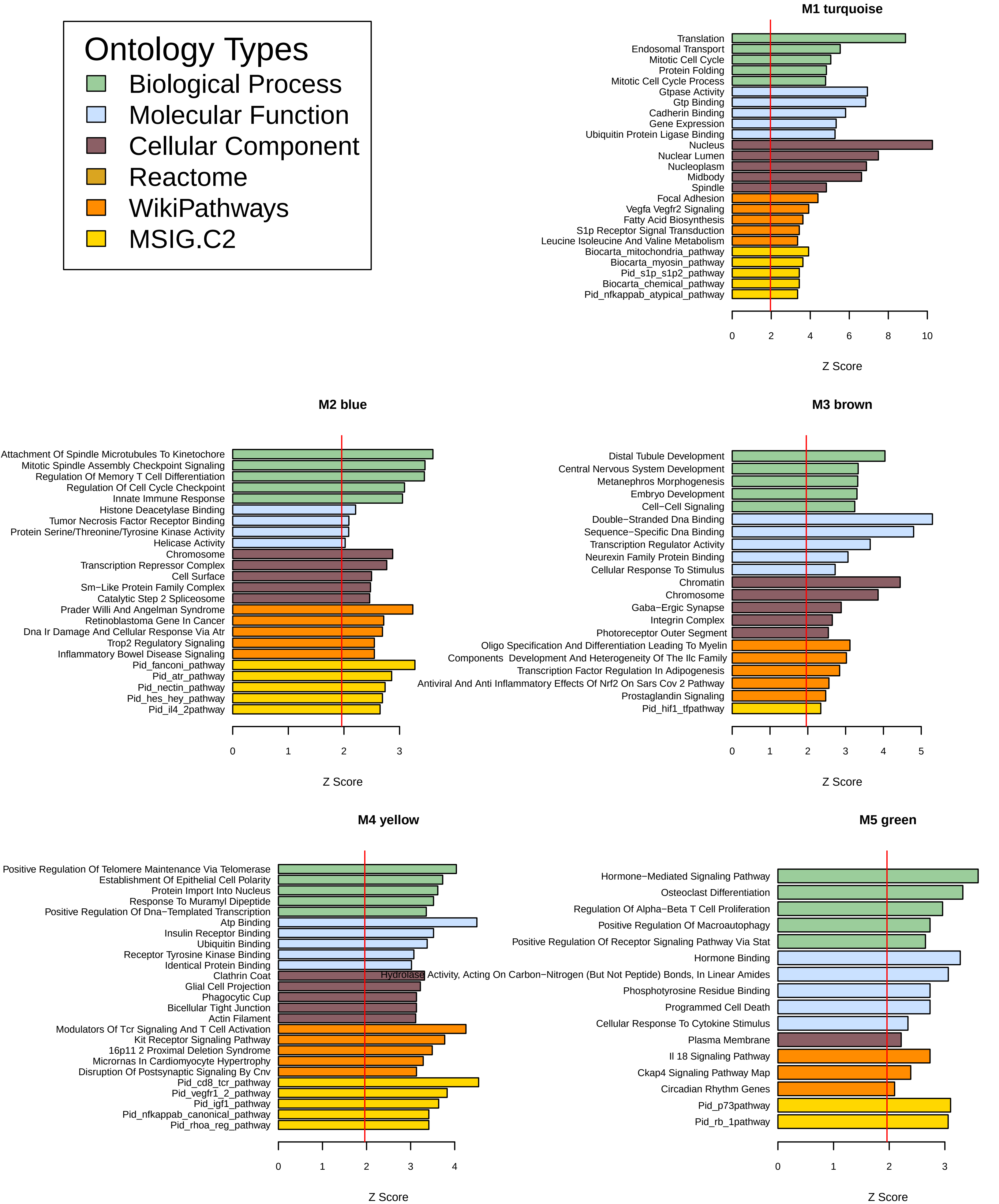

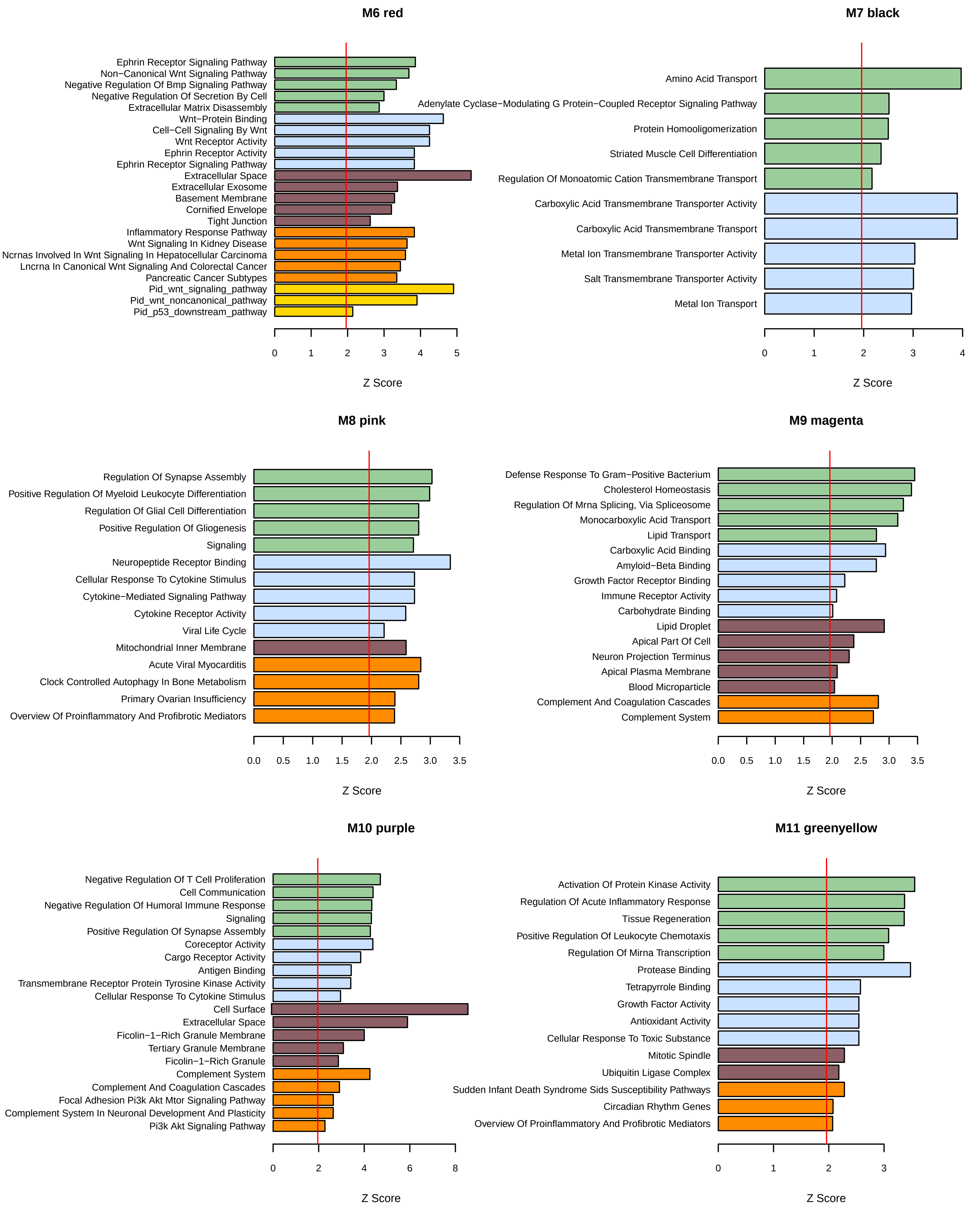
M6 red
Ephrin Receptor Signaling Pathway
Non−Canonical Wnt Signaling Pathway
Negative Regulation Of Bmp Signaling Pathway
Negative Regulation Of Secretion By Cell
Extracellular Matrix Disassembly
Wnt−Protein Binding
Cell−Cell Signaling By Wnt
Wnt Receptor Activity
Ephrin Receptor Activity
Ephrin Receptor Signaling Pathway
Extracellular Space
Extracellular Exosome
Basement Membrane
Cornified Envelope
Tight Junction
Inflammatory Response Pathway
Wnt Signaling In Kidney Disease
Ncrnas Involved In Wnt Signaling In Hepatocellular Carcinoma
Lncrna In Canonical Wnt Signaling And Colorectal Cancer
Pancreatic Cancer Subtypes
Pid_wnt_signaling_pathway
Pid_wnt_noncanonical_pathway
Pid_p53_downstream_pathway
0 1 2 3 4 5
Z Score
M7 black
Amino Acid Transport
Adenylate Cyclase−Modulating G Protein−Coupled Receptor Signaling Pathway
Protein Homooligomerization
Striated Muscle Cell Differentiation
Regulation Of Monoatomic Cation Transmembrane Transport
Carboxylic Acid Transmembrane Transporter Activity
Carboxylic Acid Transmembrane Transport
Metal Ion Transmembrane Transporter Activity
Salt Transmembrane Transporter Activity
Metal Ion Transport
0 1 2 3 4
Z Score
M8 pink
Regulation Of Synapse Assembly
Positive Regulation Of Myeloid Leukocyte Differentiation
Regulation Of Glial Cell Differentiation
Positive Regulation Of Gliogenesis
Signaling
Neuropeptide Receptor Binding
Cellular Response To Cytokine Stimulus
Cytokine−Mediated Signaling Pathway
Cytokine Receptor Activity
Viral Life Cycle
Mitochondrial Inner Membrane
Acute Viral Myocarditis
Clock Controlled Autophagy In Bone Metabolism
Primary Ovarian Insufficiency
Overview Of Proinflammatory And Profibrotic Mediators
0.0 0.5 1.0 1.5 2.0 2.5 3.0 3.5
Z Score
M9 magenta
Defense Response To Gram−Positive Bacterium
Cholesterol Homeostasis
Regulation Of Mrna Splicing, Via Spliceosome
Monocarboxylic Acid Transport
Lipid Transport
Carboxylic Acid Binding
Amyloid−Beta Binding
Growth Factor Receptor Binding
Immune Receptor Activity
Carbohydrate Binding
Lipid Droplet
Apical Part Of Cell
Neuron Projection Terminus
Apical Plasma Membrane
Blood Microparticle
Complement And Coagulation Cascades
Complement System
0.0 0.5 1.0 1.5 2.0 2.5 3.0 3.5
Z Score
M10 purple
Negative Regulation Of T Cell Proliferation
Cell Communication
Negative Regulation Of Humoral Immune Response
Signaling
Positive Regulation Of Synapse Assembly
Coreceptor Activity
Cargo Receptor Activity
Antigen Binding
Transmembrane Receptor Protein Tyrosine Kinase Activity
Cellular Response To Cytokine Stimulus
Cell Surface
Extracellular Space
Ficolin−1−Rich Granule Membrane
Tertiary Granule Membrane
Ficolin−1−Rich Granule
Complement System
Complement And Coagulation Cascades
Focal Adhesion Pi3k Akt Mtor Signaling Pathway
Complement System In Neuronal Development And Plasticity
Pi3k Akt Signaling Pathway
0 2 4 6 8
Z Score
M11 greenyellow
Activation Of Protein Kinase Activity
Regulation Of Acute Inflammatory Response
Tissue Regeneration
Positive Regulation Of Leukocyte Chemotaxis
Regulation Of Mirna Transcription
Protease Binding
Tetrapyrrole Binding
Growth Factor Activity
Antioxidant Activity
Cellular Response To Toxic Substance
Mitotic Spindle
Ubiquitin Ligase Complex
Sudden Infant Death Syndrome Sids Susceptibility Pathways
Circadian Rhythm Genes
Overview Of Proinflammatory And Profibrotic Mediators
0 1 2 3
Z Score

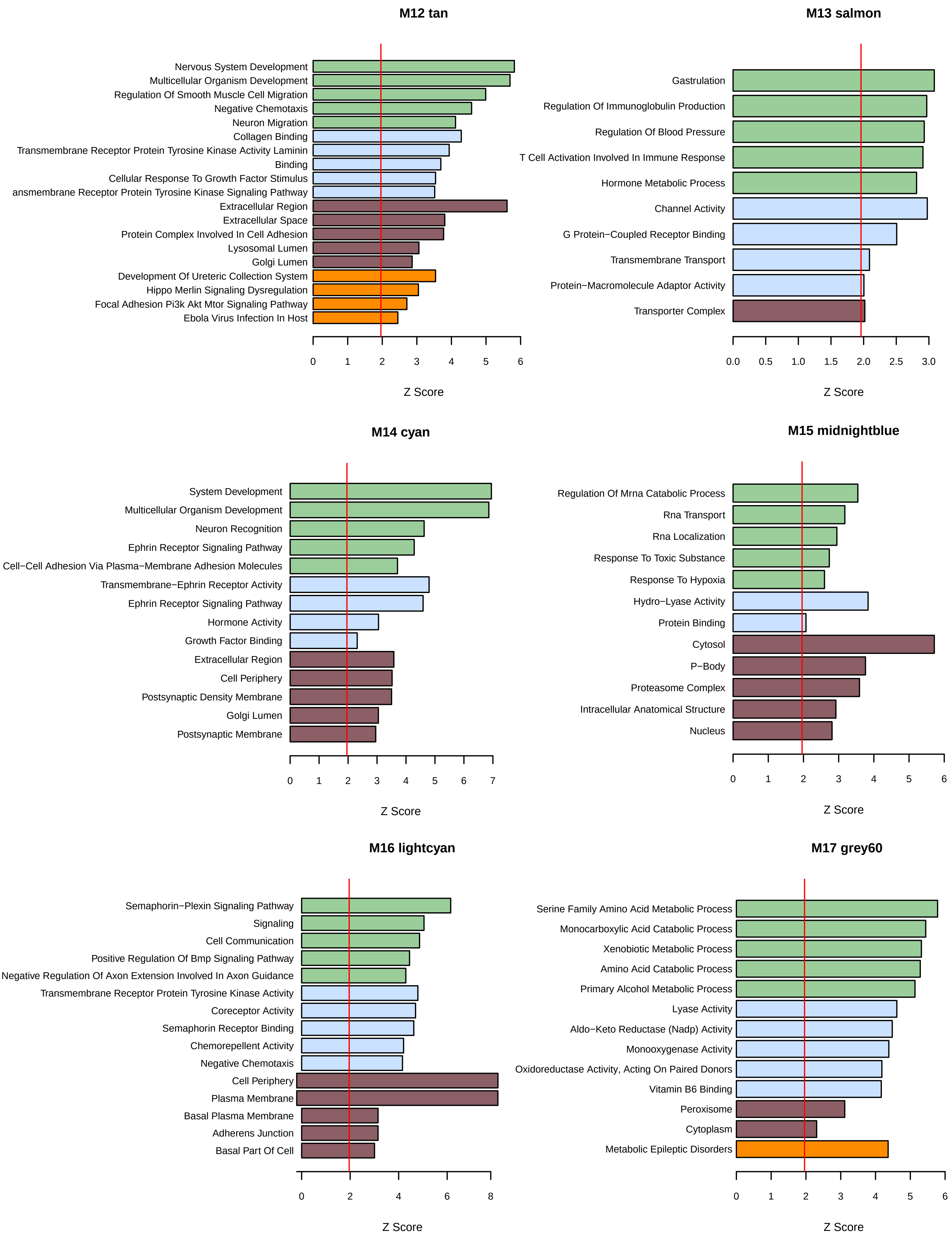

M12 tan
Nervous System Development
Multicellular Organism Development
Regulation Of Smooth Muscle Cell Migration
Negative Chemotaxis
Neuron Migration
Collagen Binding
Transmembrane Receptor Protein Tyrosine Kinase Activity Laminin
Binding
Cellular Response To Growth Factor Stimulus
ansmembrane Receptor Protein Tyrosine Kinase Signaling Pathway
Extracellular Region
Extracellular Space
Protein Complex Involved In Cell Adhesion
Lysosomal Lumen
Golgi Lumen
Development Of Ureteric Collection System
Hippo Merlin Signaling Dysregulation
Focal Adhesion Pi3k Akt Mtor Signaling Pathway
Ebola Virus Infection In Host
0 1 2 3 4 5 6
Z Score
M13 salmon
Gastrulation
Regulation Of Immunoglobulin Production
Regulation Of Blood Pressure
T Cell Activation Involved In Immune Response
Hormone Metabolic Process
Channel Activity
G Protein−Coupled Receptor Binding
Transmembrane Transport
Protein−Macromolecule Adaptor Activity
Transporter Complex
0.0 0.5 1.0 1.5 2.0 2.5 3.0
Z Score
M14 cyan
System Development
Multicellular Organism Development
Neuron Recognition
Ephrin Receptor Signaling Pathway
Cell−Cell Adhesion Via Plasma−Membrane Adhesion Molecules
Transmembrane−Ephrin Receptor Activity
Ephrin Receptor Signaling Pathway
Hormone Activity
Growth Factor Binding
Extracellular Region
Cell Periphery
Postsynaptic Density Membrane
Golgi Lumen
Postsynaptic Membrane
0 1 2 3 4 5 6 7
Z Score
M15 midnightblue
Regulation Of Mrna Catabolic Process
Rna Transport
Rna Localization
Response To Toxic Substance
Response To Hypoxia
Hydro−Lyase Activity
Protein Binding
Cytosol
P−Body
Proteasome Complex
Intracellular Anatomical Structure
Nucleus
0 1 2 3 4 5 6
Z Score
M16 lightcyan
Semaphorin−Plexin Signaling Pathway
Signaling
Cell Communication
Positive Regulation Of Bmp Signaling Pathway
Negative Regulation Of Axon Extension Involved In Axon Guidance
Transmembrane Receptor Protein Tyrosine Kinase Activity
Coreceptor Activity
Semaphorin Receptor Binding
Chemorepellent Activity
Negative Chemotaxis
Cell Periphery
Plasma Membrane
Basal Plasma Membrane
Adherens Junction
Basal Part Of Cell
0 2 4 6 8
Z Score
M17 grey60
Serine Family Amino Acid Metabolic Process
Monocarboxylic Acid Catabolic Process
Xenobiotic Metabolic Process
Amino Acid Catabolic Process
Primary Alcohol Metabolic Process
Lyase Activity
Aldo−Keto Reductase (Nadp) Activity
Monooxygenase Activity
Oxidoreductase Activity, Acting On Paired Donors
Vitamin B6 Binding
Peroxisome
Cytoplasm
Metabolic Epileptic Disorders
0 1 2 3 4 5 6
Z Score

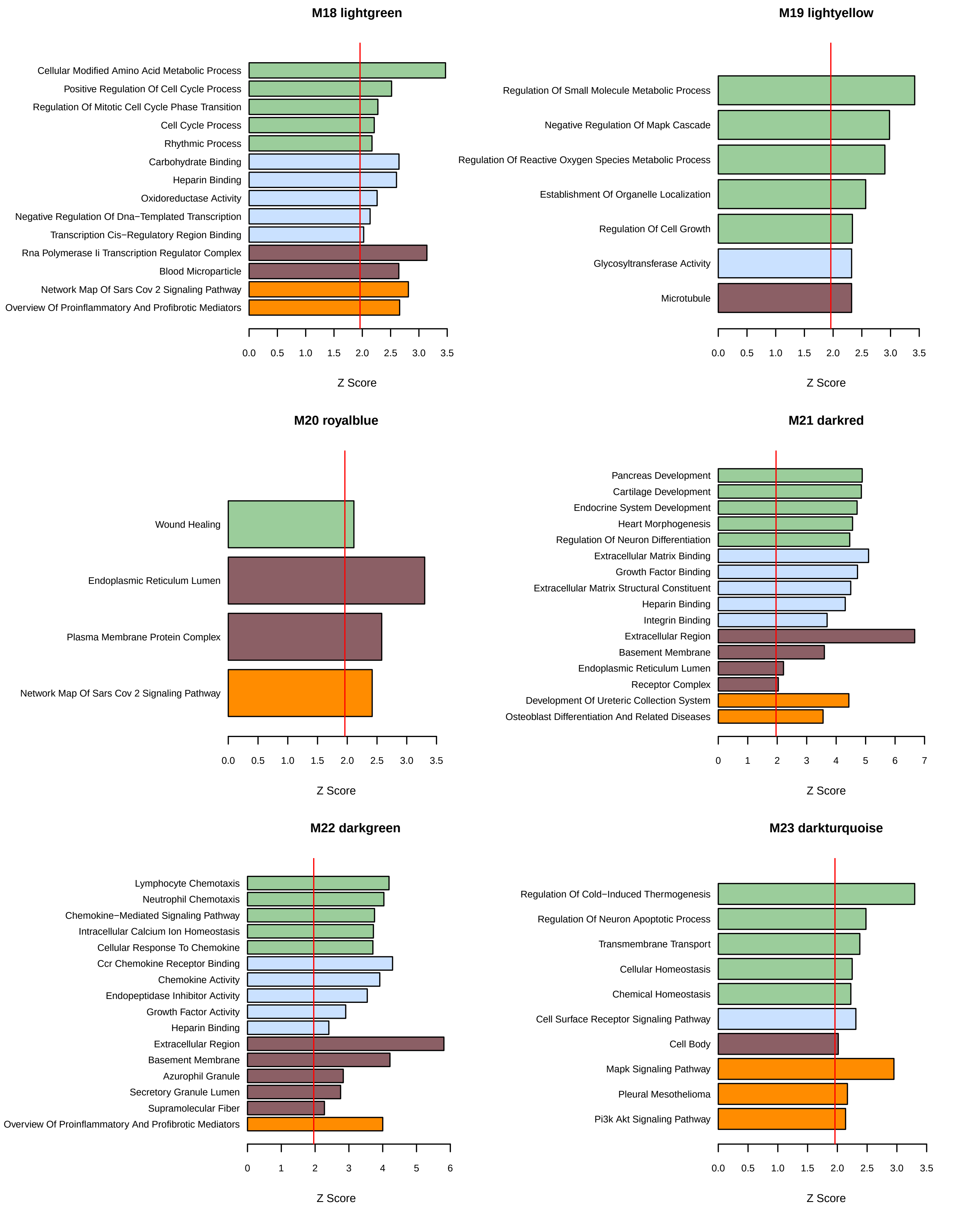

M18 lightgreen
Cellular Modified Amino Acid Metabolic Process
Positive Regulation Of Cell Cycle Process
Regulation Of Mitotic Cell Cycle Phase Transition
Cell Cycle Process
Rhythmic Process
Carbohydrate Binding
Heparin Binding
Oxidoreductase Activity
Negative Regulation Of Dna−Templated Transcription
Transcription Cis−Regulatory Region Binding
Rna Polymerase Ii Transcription Regulator Complex
Blood Microparticle
Network Map Of Sars Cov 2 Signaling Pathway
Overview Of Proinflammatory And Profibrotic Mediators
0.0 0.5 1.0 1.5 2.0 2.5 3.0 3.5
Z Score
M19 lightyellow
Regulation Of Small Molecule Metabolic Process
Negative Regulation Of Mapk Cascade
Regulation Of Reactive Oxygen Species Metabolic Process
Establishment Of Organelle Localization
Regulation Of Cell Growth
Glycosyltransferase Activity
Microtubule
0.0 0.5 1.0 1.5 2.0 2.5 3.0 3.5
Z Score
M20 royalblue
Wound Healing
Endoplasmic Reticulum Lumen
Plasma Membrane Protein Complex
Network Map Of Sars Cov 2 Signaling Pathway
0.0 0.5 1.0 1.5 2.0 2.5 3.0 3.5
Z Score
M21 darkred
Pancreas Development
Cartilage Development
Endocrine System Development
Heart Morphogenesis
Regulation Of Neuron Differentiation
Extracellular Matrix Binding
Growth Factor Binding
Extracellular Matrix Structural Constituent
Heparin Binding
Integrin Binding
Extracellular Region
Basement Membrane
Endoplasmic Reticulum Lumen
Receptor Complex
Development Of Ureteric Collection System
Osteoblast Differentiation And Related Diseases
0 1 2 3 4 5 6 7
Z Score
M22 darkgreen
Lymphocyte Chemotaxis
Neutrophil Chemotaxis
Chemokine−Mediated Signaling Pathway
Intracellular Calcium Ion Homeostasis
Cellular Response To Chemokine
Ccr Chemokine Receptor Binding
Chemokine Activity
Endopeptidase Inhibitor Activity
Growth Factor Activity
Heparin Binding
Extracellular Region
Basement Membrane
Azurophil Granule
Secretory Granule Lumen
Supramolecular Fiber
Overview Of Proinflammatory And Profibrotic Mediators
0 1 2 3 4 5 6
Z Score
M23 darkturquoise
Regulation Of Cold−Induced Thermogenesis
Regulation Of Neuron Apoptotic Process
Transmembrane Transport
Cellular Homeostasis
Chemical Homeostasis
Cell Surface Receptor Signaling Pathway
Cell Body
Mapk Signaling Pathway
Pleural Mesothelioma
Pi3k Akt Signaling Pathway
0.0 0.5 1.0 1.5 2.0 2.5 3.0 3.5
Z Score

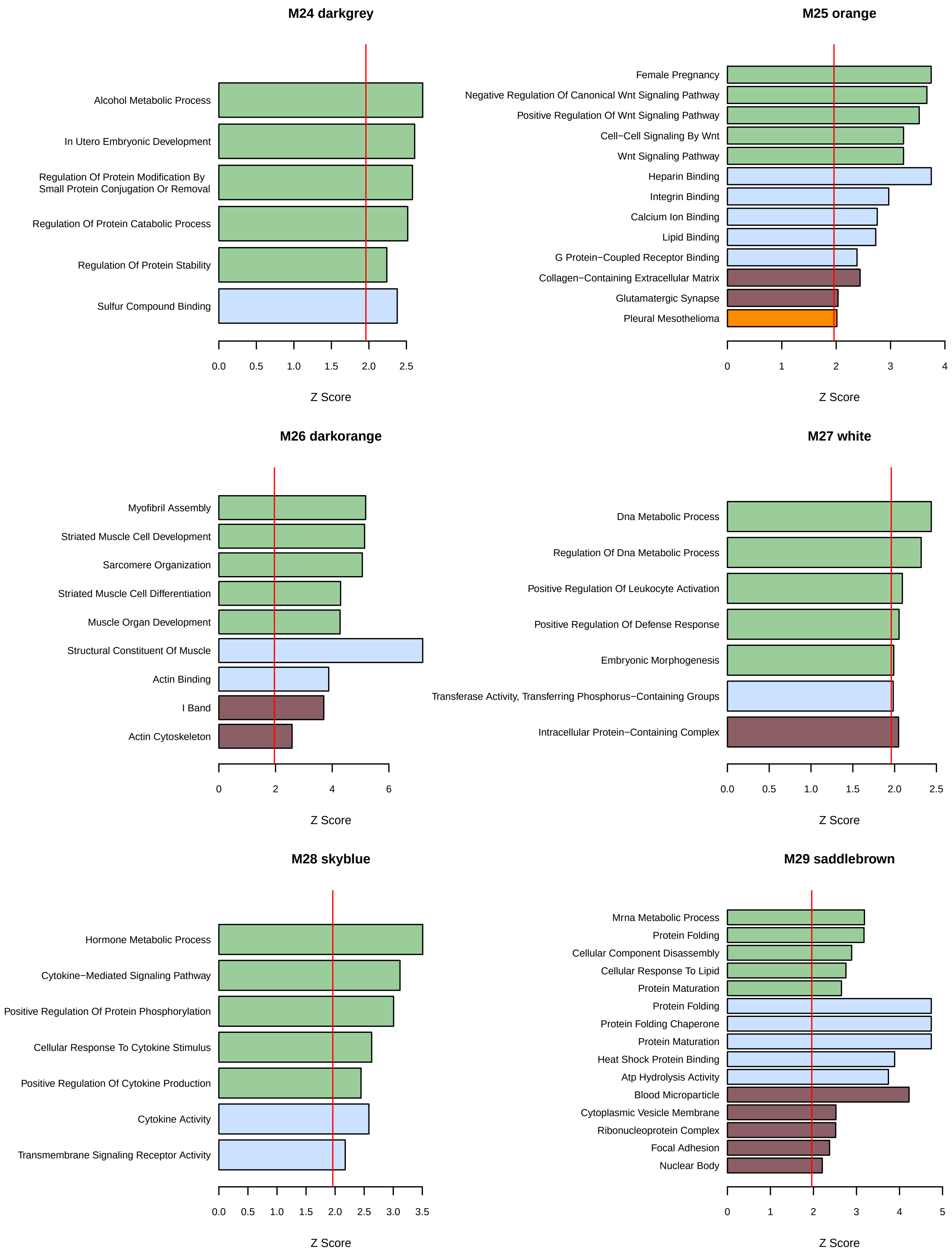
M24 darkgrey
Alcohol Metabolic Process
In Utero Embryonic Development
Regulation Of Protein Modification By
Small Protein Conjugation Or Removal
Regulation Of Protein Catabolic Process
Regulation Of Protein Stability
Sulfur Compound Binding
0.0
0.5
1.0
1.5
2.0
2.5
Z Score
M25 orange
Female Pregnancy
Negative Regulation Of Canonical Wnt Signaling Pathway
Positive Regulation Of Wnt Signaling Pathway
Cell−Cell Signaling By Wnt
Wnt Signaling Pathway
Heparin Binding
Integrin Binding
Calcium Ion Binding
Lipid Binding
G Protein−Coupled Receptor Binding
Collagen−Containing Extracellular Matrix
Glutamatergic Synapse
Pleural Mesothelioma
0
1
2
3
4
Z Score
M26 darkorange
Myofibril Assembly
Striated Muscle Cell Development
Sarcomere Organization
Striated Muscle Cell Differentiation
Muscle Organ Development
Structural Constituent Of Muscle
Actin Binding
I Band
Actin Cytoskeleton
0
2
4
6
Z Score
M27 white
Dna Metabolic Process
Regulation Of Dna Metabolic Process
Positive Regulation Of Leukocyte Activation
Positive Regulation Of Defense Response
Embryonic Morphogenesis
Transferase Activity, Transferring Phosphorus−Containing Groups
Intracellular Protein−Containing Complex
0.0
0.5
1.0
1.5
2.0
2.5
Z Score
M28 skyblue
Hormone Metabolic Process
Cytokine−Mediated Signaling Pathway
Positive Regulation Of Protein Phosphorylation
Cellular Response To Cytokine Stimulus
Positive Regulation Of Cytokine Production
Cytokine Activity
Transmembrane Signaling Receptor Activity
0.0
0.5
1.0
1.5
2.0
2.5
3.0
3.5
Z Score
M29 saddlebrown
Mrna Metabolic Process
Protein Folding
Cellular Component Disassembly
Cellular Response To Lipid
Protein Maturation
Protein Folding
Protein Folding Chaperone
Protein Maturation
Heat Shock Protein Binding
Atp Hydrolysis Activity
Blood Microparticle
Cytoplasmic Vesicle Membrane
Ribonucleoprotein Complex
Focal Adhesion
Nuclear Body
0
1
2
3
4
5
Z Score

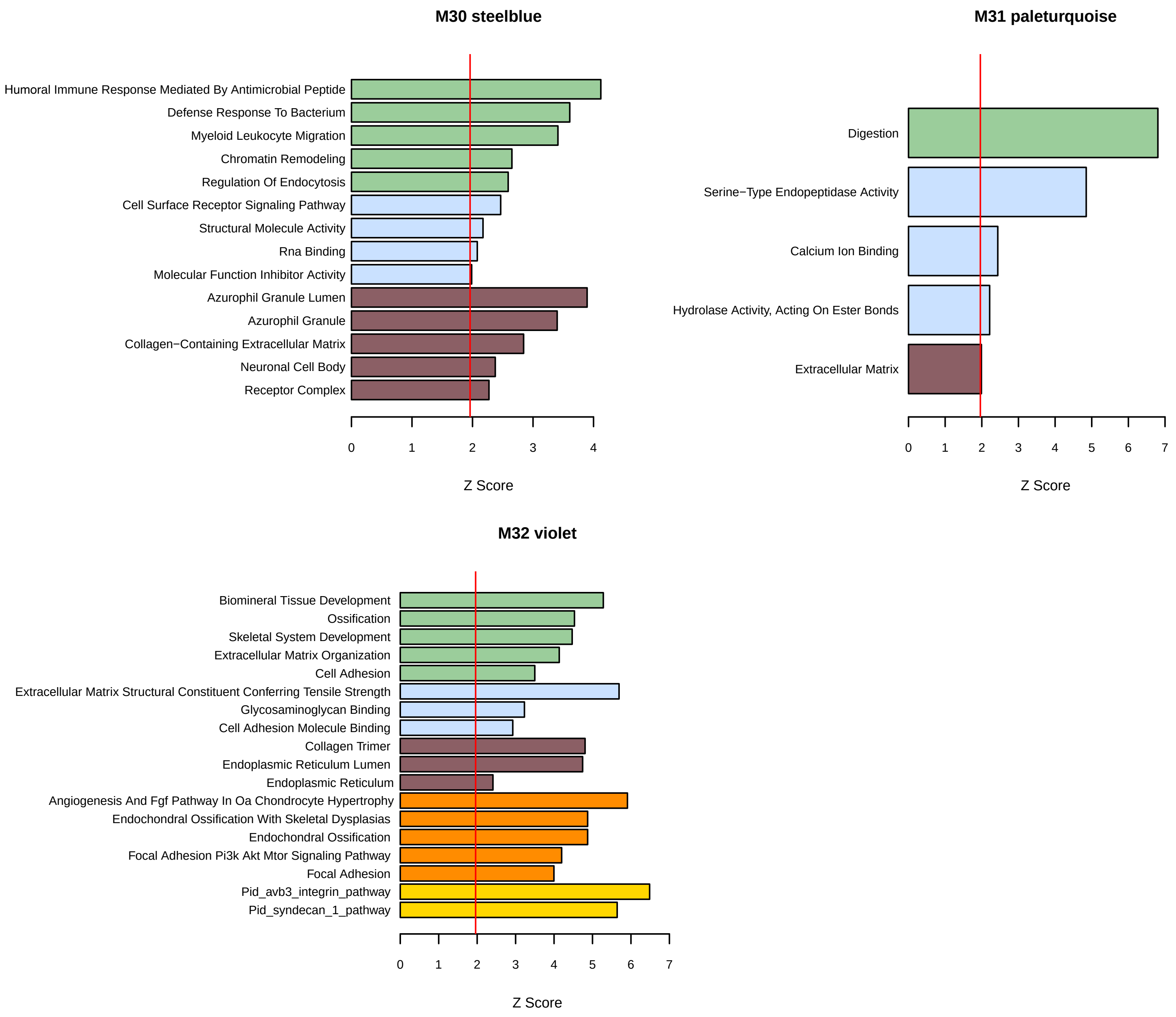
M30 steelblue
Humoral Immune Response Mediated By Antimicrobial Peptide
Defense Response To Bacterium
Myeloid Leukocyte Migration
Chromatin Remodeling
Regulation Of Endocytosis
Cell Surface Receptor Signaling Pathway
Structural Molecule Activity
Rna Binding
Molecular Function Inhibitor Activity
Azurophil Granule Lumen
Azurophil Granule
Collagen−Containing Extracellular Matrix
Neuronal Cell Body
Receptor Complex
0 1 2 3 4
Z Score
M31 paleturquoise
Digestion
Serine−Type Endopeptidase Activity
Calcium Ion Binding
Hydrolase Activity, Acting On Ester Bonds
Extracellular Matrix
0 1 2 3 4 5 6 7
Z Score
M32 violet
Biomineral Tissue Development
Ossification
Skeletal System Development
Extracellular Matrix Organization
Cell Adhesion
Extracellular Matrix Structural Constituent Conferring Tensile Strength
Glycosaminoglycan Binding
Cell Adhesion Molecule Binding
Collagen Trimer
Endoplasmic Reticulum Lumen
Endoplasmic Reticulum
Angiogenesis And Fgf Pathway In Oa Chondrocyte Hypertrophy
Endochondral Ossification With Skeletal Dysplasias
Endochondral Ossification
Focal Adhesion Pi3k Akt Mtor Signaling Pathway
Focal Adhesion
Pid_avb3_integrin_pathway
Pid_syndecan_1_pathway
0 1 2 3 4 5 6 7
Z Score
